# Spectral comb of highly chirped pulses generated via cascaded FWM of two frequency-shifted dissipative solitons

**DOI:** 10.1038/s41598-017-03092-2

**Published:** 2017-06-06

**Authors:** Evgeniy V. Podivilov, Denis S. Kharenko, Anastasia E. Bednyakova, Mikhail P. Fedoruk, Sergey A. Babin

**Affiliations:** 1 Institute of Automation and Electrometry, SB RAS, Novosibirsk, 630090 Russia; 2 Novosibirsk State University, Novosibirsk, 630090 Russia; 3 Institute of Computational Technologies, SB RAS, Novosibirsk, 630090 Russia

## Abstract

Dissipative solitons generated in normal-dispersion mode-locked lasers are stable localized coherent structures with a mostly linear frequency modulation (chirp). The soliton energy in fiber lasers is limited by the Raman effect, but implementation of the intracavity feedback at the Stokes-shifted wavelength enables synchronous generation of a coherent Raman dissipative soliton. Here we demonstrate a new approach for generating chirped pulses at new wavelengths by mixing in a highly-nonlinear fiber of these two frequency-shifted dissipative solitons, as well as cascaded generation of their clones forming in the spectral domain a comb of highly chirped pulses. We observed up to eight equidistant components in the interval of more than 300 nm, which demonstrate compressibility from ~10 ps to ~300 fs. This approach, being different from traditional frequency combs, can inspire new developments in fundamental science and applications such as few-cycle/arbitrary-waveform pulse synthesis, comb spectroscopy, coherent communications and bio-imaging.

## Introduction

It is well known that the phase synchronization of laser modes (so-called mode locking) forms a pulse train with a period equal to the inverse mode spacing and duration equal to the inverse gain bandwidth^1^. In the case of a broadband gain medium, the mode-locked laser can generate ultrashort pulses while its broad spectrum consisting of equidistant frequencies represents so-called “frequency comb”^2, 3^. In Ti:sapphire lasers, the comb width can reach one octave^4^. Such a broad coherent spectrum usually called “supercontinuum” (SC) has opened a new area of optics – frequency metrology^2^.

A coherent SC can also be generated outside the laser cavity, e.g. by launching the mode-locked laser pulse (that can form a soliton in anomalous-dispersion regime) into a highly nonlinear medium, either bulk crystals/silica^5^ or silica-based fiber waveguides, in particular, tapered ones or photonic-crystal fibers (PCF) characterized by high nonlinearity and controllable dispersion^6^. The effects induced by the Kerr nonlinearity, such as self-phase modulation (SPM) or cross-phase modulation (XPM), broaden the spectrum, while the increasing group velocity dispersion limits the SC bandwidth^6–9^. The Kerr nonlinearity effects for multi-frequency radiation can be treated in the frequency domain as cascaded four-wave mixing (FWM) between corresponding modes/frequencies^8, 9^ which results in generation of additional spectral components, thus forming a coherent comb. Kerr frequency combs can be also generated via FWM parametric oscillation in microresonators^10^, which are especially attractive for mid-IR range^11, 12^. The technique of microresonators also enables mode locking and soliton formation^12, 13^. Simultaneous second- and third- harmonic conversion of a Kerr comb within the same microresonator leads to generation of comb-like visible lines^14, 15^.

Dissipative solitons (DS) are known as stable localized coherent structures formed due to a balance between loss and gain, nonlinearity and dispersion. In contrast to conservative solitons, DSs generally have a fixed profile, and distinct dynamical features, such as pulsations, bifurcations, soliton molecule formation etc.^16^. The concept of dissipative solitons provides an excellent framework for understanding complex pulse dynamics in passively mode-locked lasers and stimulates development of innovative cavity designs. The pulses generated in normal-dispersion mode-locked fiber lasers are usually DS characterized by high linear frequency modulation (chirp) along the pulse^16, 17^. As shown recently, Stokes-shifted Raman dissipative solitons (RDS) can be synchronously generated in the same laser cavity^18, 19^. In this paper, we study the mixing between these nearly identical coherent chirped pulses with different carrier frequencies in a highly nonlinear medium (PCF as an example). It appears that multiple equidistant chirped pulse lines are generated at harmonics of the DS-RDS frequency difference both in long- and short-wavelength spectral domains thus forming a “comb of chirped pulses”. The theoretical background, proof-of-principle experiments and potential applications of this new approach are described below.

## Results

Let us treat two (±) coherent pulses with amplitude *A*_±_(*t*) and carrier frequency *ω*_±_. The amplitude of the combined field is:

1 $$E(t)={A}_{+}(t)\exp (i{\omega }_{+}t)+{A}_{-}(t)\exp (i{\omega }_{-}t)$$

Assume that the frequency difference *Δ*_*st*_ = *ω*_+_−*ω*_−_ is much larger than the pulse spectral width *Δ*_±_. Launching the pulses into a PCF having zero dispersion near *ω*_±_ will result in generation of new spectral components at *ω*_*1*±_ = *ω*_±_ ± *Δ*_*st*_ via the FWM process. Their amplitudes *A*_*1*±_ grow linearly with distance *z* = *ct*/*n*:

2 $${A}_{1\pm }(t)=i\gamma z{A}_{\pm }^{2}(t){A}_{\mp }^{\ast }(t),$$

where *γ* is the Kerr nonlinearity coefficient. Similar four-wave model was used e.g. for modeling of micro-resonators with CW bi-chromatic pumping^20^.

**Figure 1:**
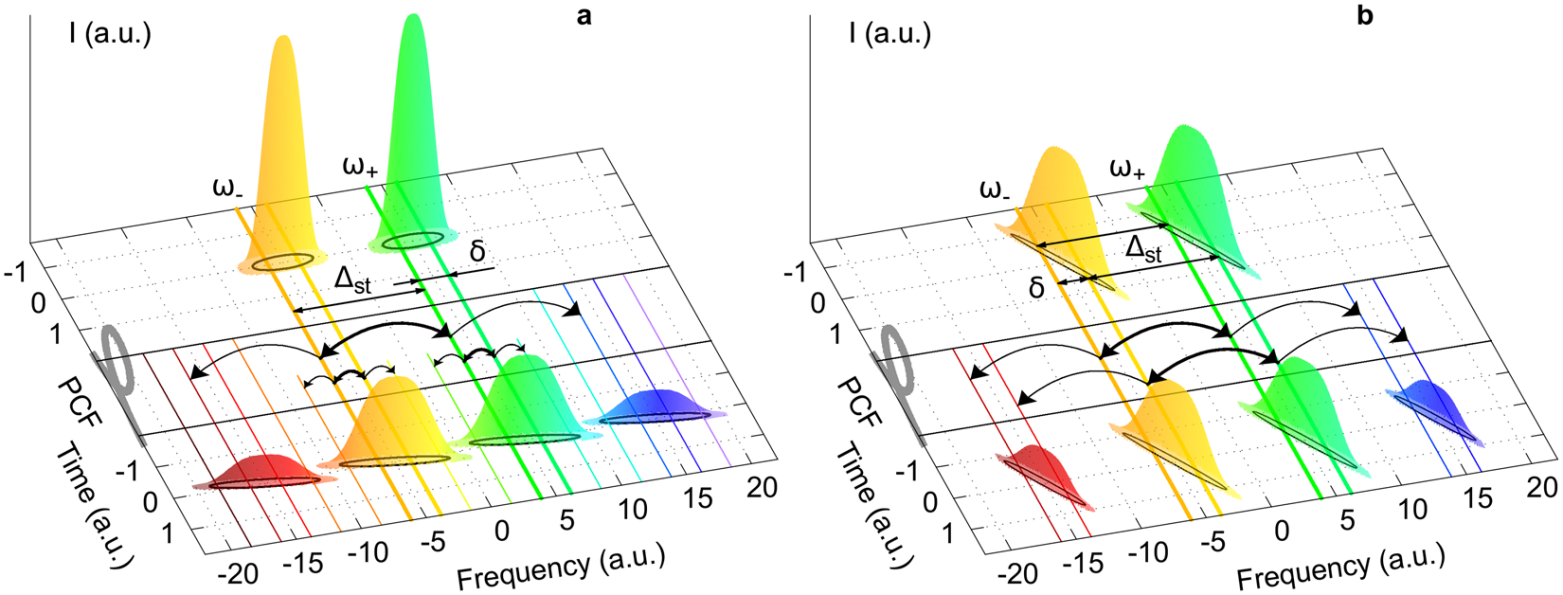
Nonlinear mixing in a PCF of two solitons (**a**) and two equally chirped dissipative solitons (**b**) with frequency separation *Δ*_*st*_, each of which consisting of laser modes with separation *δ*.

In the first perturbation order, the spectral satellite power *P*_*1*±_ = *A*_*1*±_^2^ is lower than the pump power *P*_±_ by factor *ρ* = *γ* ^*2*^ *z* ^*2*^ *P*_+_ *P*_−_ ≪ 1. Increasing satellite amplitude *A*_*1*±_ initiates FWM between the satellites and pump pulses thus resulting in generation of higher-order components *ω*_±_ ± *2Δ*_*st*_ with the power lower by *ρ* ^*2*^/*2* times as compared to the pump power. Herewith, propagation of the pump pulses is accompanied by the variation of their phase via the SPM and XPM processes as ${A}_{\pm }^{out}={A}_{\pm }(t)\exp [i\gamma z({P}_{\pm }(t)+2{P}_{\mp }(t))]$. For transform limited pulses (e.g. solitons) with temporal and spectral half-widths *T* and *Δ* satisfying to *T∙Δ*~1, the relative spectral broadening is *δΔ*/*Δ~3ρ*. The corresponding qualitative picture in the time-frequency plane is shown in Fig. 1а. The process of spectral broadening can be represented as FWM between internal frequency components (laser modes) with spacing *δ* resulting in generation of new components separated by *δ*. When we mix two mutually coherent solitons with frequency separation *Δ*_*st*_, which overlap in time, additional components separated by *Δ*_*st*_ appear after passing PCF. The processes of new components generation (~*Δ*_*st*_) and pulse broadening (~*δ*) occur simultaneously (Fig. 1a), finally leading to the formation of a continuously broadened frequency comb.

For chirped dissipative solitons of the same spectral width *Δ*, the picture is principally different. The DS features an increased time-bandwidth product, *T∙Δ~f* ≫ 1, as the frequency chirp stretches the pulse along the diagonal of the *Δ-T* square (Fig. 1b). If two input pulses with carrier frequencies *ω*_±_ have a similar chirp parameter *f*, the frequency spacing *Δ*_*st*_ = *ω*_+_ − *ω*_−_ remains unchanged from the front to the trailing edge of the pulses, when they propagate in the low-dispersion window of PCF. Therefore, satellites at *ω*_±_ ± *Δ*_*st*_ generated in the FWM process should be also chirped. Let us treat two Gaussian input pulses ${A}_{\pm }(t)={A}_{\pm }\exp (-{t}^{2}(1+i{f}_{\pm })/2{T}_{\pm }^{2})$ with a corresponding half-width *T*_±_ and chirp parameter *f*_±_ ≫ 1. Their spectrum is also Gaussian: ${I}_{\pm }(\omega )\propto \exp (-{(\omega -{\omega }_{\pm })}^{2}{/{\rm{\Delta }}}_{\pm }^{2})$, where ${{\rm{\Delta }}}_{\pm }={(1+{f}_{\pm }^{2}/{T}_{\pm }^{2})}^{1/2}\approx {f}_{\pm }/{T}_{\pm }$ is the spectral half-width. According to (2), satellites born in the FWM process have duration ${T}_{1\pm }={({T}_{\pm }^{-2}+2{T}_{\mp }^{-2})}^{-1/2}$, chirp parameter ${f}_{1\pm }=(2{f}_{\pm }{T}_{\pm }^{-2}-{f}_{\mp }{T}_{\mp }^{-2}){T}_{1\pm }^{2}$ and spectral width ${{\rm{\Delta }}}_{1\pm }\approx {f}_{1\pm }/{T}_{1\pm }$. In the case of two identical pump pulses, the satellites temporal/spectral widths are reduced by factor $\sqrt{3}$ in the first perturbation order. At the same time, the SPM/XPM-induced spectral broadening *δΔ*~*3ρ*/*T* is small relative to the linewidth, *δΔ*/*Δ~3ρ*/*f*, as *f* ≫ 1. In terms of the FWM process, it means that the mixing between intra-pulse components *δ* is suppressed because they do not overlap in time (see Fig. 1b).

With increasing length and/or power, parameter *ρ* grows up to unity and multiple lines in the low-dispersion spectral window should be generated with the same amplitude and width, i.e. a comb of similar chirped pulses will arise. However, representation of nonlinear process as SPM, XPM, FWM is not possible at high powers, so a more general description is necessary in this case. It was developed for identical input pulses both analytically and numerically, see Methods section. The models give close results in zero-dispersion case, whereas numerical simulation allows to include into consideration the dispersion and Raman processes, which are important in the experiment.

Let us mention that the cascaded FWM process with bi-chromatic pulsed pumping has been experimentally studied in^21^, where new narrowband spectral components are observed, whose number was a function of the frequency spacing between two input pulses. However, internal structure of each spectral component and influence of the input pulse chirping have not been studied in that paper. Here we explore, for the first time to our knowledge, the mixing of highly-chirped dissipative solitons studying the internal structure of the generated spectral components not only in spectral, but also in time domain thus demonstrating a possibility to generate a comb of similar highly chirped pulses.

**Figure 2:**
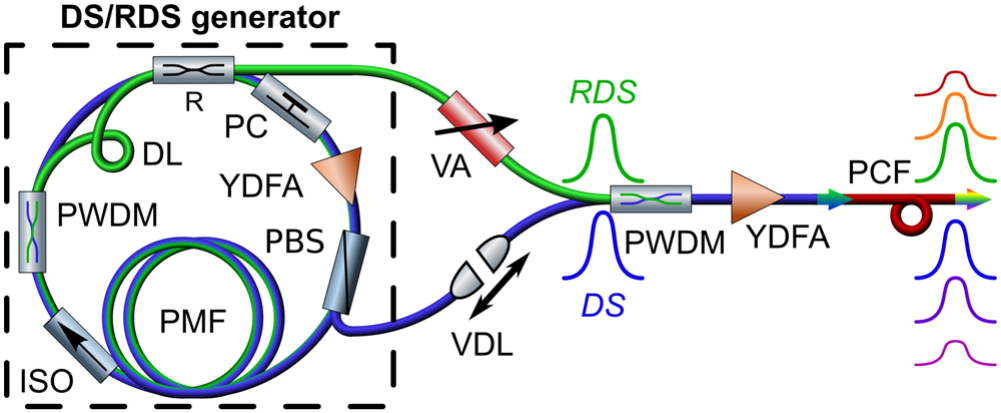
Experimental setup: The DS/RDS generator has two fiber outputs for the DS and RDS, which are combined by polarization wavelength division multiplexer (PWDM) after temporal and amplitude equalization by a variable delay line (VDL) and a variable attenuator (VA). After amplification by Yb-doped fiber (YDFA) they propagate together in the PCF thus generating their clones at new wavelengths both in short (anti-Stokes) and long (Stokes) wavelength domains. All components are PM, except for the intra-cavity YDFA with pigtails and the PCF.

To perform a proof-of-principle experiment the following setup was assembled, see Fig. 2. We test a 1-meter PCF (SC-5.5-1040) with a zero dispersion wavelength (ZDW) of 1040 nm and two chirped pulses with wavelengths 1025 and 1070 nm generated in a common cavity of a DS/RDS generator^18, 19^ (see Methods section for details). The DS and RDS generated in a common cavity are shown to be mutually coherent^19^, that is a question in case of external-cavity RDS generation demonstrated recently for fiber and diamond Raman lasers^22, 23^. After matching the DS and RDS with a variable delay line (VDL) and equalization with a variable attenuator (VA), the mutually coherent chirped pulses are combined with proper polarization at the external coupler (PWDM) and are launched into the PCF via loss-compensating Yb-doped fiber amplifier (YDFA). The mixed pulses have duration of about 20 ps and total energy up to 16 nJ.

**Figure 3:**
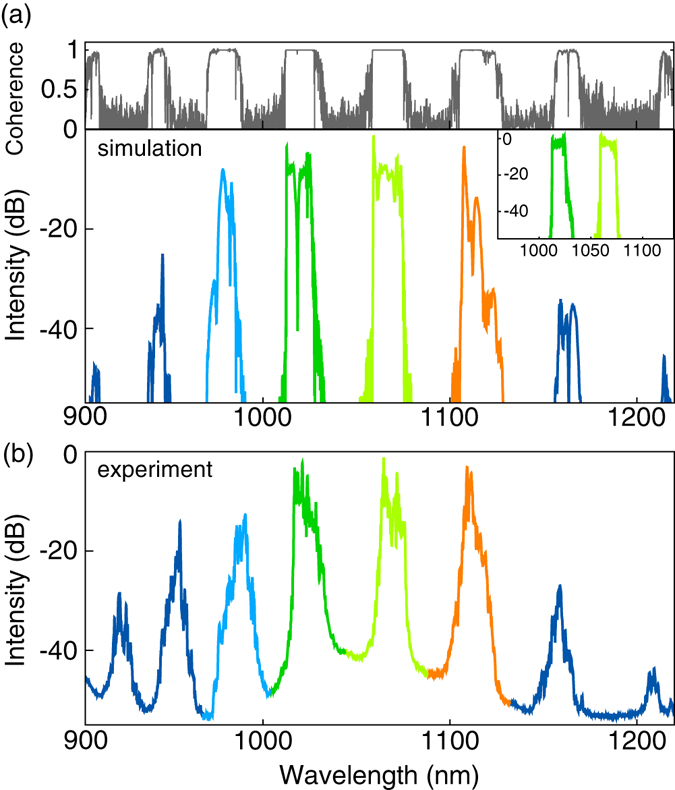
Output spectra in simuation (**a**) and experiment (**b**). Inset: spectral shape of the DS and RDS at the input of the nonlinear fiber. Upper panel: coherence degree for corresponding spectral components calculated by the method described in^24^.

At the PCF output, new equidistant spectral components are observed, as predicted by the simulation under conditions of the experiment (see Fig. 3). The initial field at the PCF input, shown in the inset of Fig. 3a, is taken from the DS/RDS fiber laser model^18, 19^ for the pulse energy equally divided between the DS and RDS (8 nJ each). The output spectrum presented in Fig. 3a is calculated numerically for the experimental conditions with account for the dispersion and Raman effects in the PCF (see Methods section). New spectral components appear at >1100 nm and <1000 nm, the most eminent of which are the first Stokes and anti-Stokes pulses at 1110 and 980 nm with 1.6 and 1.7 nJ energies, respectively. At that, the DS and RDS pump pulses are noticibly depleted at the output due to the energy transfer to the Stokes and anti-Stokes components. One can also measure numerically the coherence degree of the output signal after propagation in the nonlinear fiber in presence of dispersion and Raman effects. The simulations for the DS and RDS pulses described above were repeated under identical conditions except for the initial random noise present in the input field. Then the coherence degradation was quantified by calculating the degree of first-order coherence as a function of wavelength across the spectrum^24^. The resulting spectrum of coherence degree, shown in upper panel of Fig. 3a, demonstrates negligibly small spectral phase fluctuations and hence high coherence of the pulses generated at new wavelengths.

**Figure 4:**
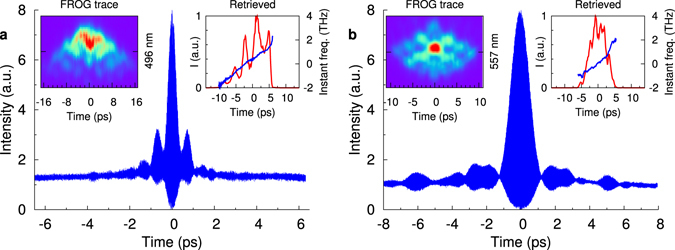
Temporal characteristics of the generated anti-Stokes (**a**) and Stokes (**b**) pulses: Interferometric ACF for a compressed pulse, FROG trace (left inset) and retrieved pulse amplitude and instantaneous frequency (right inset).

The experimental spectra (Fig. 3b) as well as the estimated total energy of satellites (2–4 nJ depending on adjustments at the input) are quite similar to those in calculations. We also characterized each satellite in more detail (see Methods section). The results are shown in Fig. 4a,b for the first anti-Stokes (~990 nm) and Stokes (~1110 nm) satellites demonstrating nearly a linear chirp within its spectral width of 10–15 nm at a duration of 10–15 ps compressible to 300–800 fs, similar to the input pulses whose parameters were measured in the experimental conditions as described in refs 18, 19: linewidth of ~15 nm and duration of ~20 ps dechirped to 230–400 fs. In spite of some difference in values, the satellites can be treated as clones of the input dissipative solitons having nearly the same chirp, whereas their number is limited by dispersion.

## Discussion

Thus, we have proposed and demonstrated theoretically and experimentally a new approach for generating chirped pulses at new wavelengths by mixing of two frequency-shifted dissipative solitons in a highly-nonlinear fiber, as well as cascaded generation of their clones. Multiple clones are separated in frequency by the same interval and have similar characteristics (spectrum, duration, and chirp) as input DSs thus forming a comb of highly-chirped pulses. The energy of an individual component reaches 4 nJ for ~8 nJ pump pulses and the compressed duration approaches ~300 fs. Up to eight ~10-nm wide chirped components in a >300-nm spectral interval are observed in the experiment with a short (1-m) PCF with rater low group velocity dispersion. Note that the mixing of chirped pulses in a long PCF with high integral dispersion is defined by the phase matching condition enabling generation of only one narrow (~1 nm) Stokes component tunable within a ~20-nm range by varying the delay between the input pulses^25^. A short PCF placed in a cavity of optical parametric amplifier or oscillator also generates only one component (Stokes or anti-Stokes), see e.g.^26^ and citation therein.

Further increase of input pulse energies and/or using a PCF with a broader low-dispersion window may result in an octave-spanning comb of equidistant chirped pulses. The comb period may be adjusted by means of the frequency difference variation between the DS and RDS or doubling it in the second-order RDS scheme^27^. This approach can be also transferred to other spectral regions by using other types of fiber lasers for generation of DS/RDS pump pulses, e.g. Er (1.55 μm) or Tm, Ho (2 μm)^28^, as well as to microresonators, which also exhibit different Raman effects such as Raman self-frequency shift of Kerr solitons^29^, Raman frequency comb formation^30^, and synchronous Stokes soliton generation in presence of the main soliton^31^.

Together with the input to fundamental science, the proposed approach offers quite new opportunities for applications. A proper phase correction and coherent combination of the comb components may be used for generation of high-energy few-cycle pulses and/or for arbitrary waveform synthesis, analogues to that with the conventional continuous frequency comb (coherent supercontinuum)^32^ or with several synchronized mode-locked lasers operating in different spectral ranges^33^. Compared to independent sources, the approach of the coherent chirped-pulses comb is intrinsically stable and much simpler in realization. Such comb centered at 1.55 micron can be implemented in ultra-broadband transmission lines with new coherent modulation/demodulation formats^34^. Other applications can also benefit from this approach, including frequency comb spectroscopy^35^, coherent biomedical imaging and microscopy^26^, mid-IR and THz generation^36^ and others.

## Methods

### Analytical model

For the sake of simplicity, we treat identical input pulses with amplitudes ${A}_{+}(t)={A}_{-}(t)=A(t)$ and carrier frequency difference *Δ*_*st*_ = *ω*_+_−*ω*_−_, then the envelope of the combined field is $B(t)=2A(t)\cos ({{\rm{\Delta }}}_{st}/2)$. Neglecting dispersion, the combined pulse envelope evolves according to equation:

3 $$\frac{\partial B}{\partial z}-\frac{1}{c}\frac{\partial B}{\partial t}=i\gamma |B{|}^{2}B,$$

Its solution at the output of the fiber of length *L* is

4 $$\begin{array}{rcl}{B}^{out}(t) & = & B(t)\exp [2i\gamma LI(t)(1+\,\cos ({{\rm{\Delta }}}_{st}t))]\\  & = & A{e}^{2\gamma LI(t)}\sum _{n=-\infty }^{\infty }\exp (in{{\rm{\Delta }}}_{st}t){i}^{n}[{J}_{n}(2\gamma LI(t))+i{J}_{n+1}(2\gamma LI(t))],\end{array}$$

where *I*(*t*) = |A(*t*)|^2^ is the intensity of each input pulse. The last equation shows that the output signal represents a “comb” of equidistant spectral components separated by *Δ*_*st*_. A satellite with number *n* takes the form of the input signal *A*(*t*) multiplied by the sum of two Bessel functions *J*_*n*_, *J*_*n*+*1*_. Let us now take the input pulses in the form of a highly chirped dissipative soliton (solution of the Ginzburg-Landau equation)^16, 37^ $A(t)={A}_{0}{\cosh }^{(if-1)}(t/T)$, where *T* is the half-width, *f* ≫ 1 is the chirp parameter, and *A*_*0*_ is the peak amplitude. Its Fourier transform is expressed via the *β* function. However in the high-chirp limit (*f* ≫ 1) the analytical expression can be obtained^38^. The output spectrum of the n-th satellite takes the following form:

5 $${P}_{n}({\rm{\Omega }})=P({J}_{n}^{2}(2\gamma L{I}_{0}(1-\frac{{{\rm{\Omega }}}^{2}}{{{\rm{\Delta }}}^{2}}))+{J}_{n+1}^{2}(2\gamma L{I}_{0}(1-\frac{{{\rm{\Omega }}}^{2}}{{{\rm{\Delta }}}^{2}}))),$$

where *Ω* is the frequency detuning, and *Δ* = *f*/*T* is the pulse spectrum half-width. For high-intensity input pulses, the amplitudes of far (large n) satellites increase, while the spectral profiles of near satellites become disturbed.

### Numerical model

The signal evolution inside the nonlinear fiber is described by the generalized nonlinear Schrὂdinger equation (NLSE):

6 $$\frac{\partial A}{\partial z}=\sum _{i=2}^{5}\frac{{i}^{k+1}}{k!}{\beta }_{k}\frac{{\partial }^{k}A}{\partial {t}^{k}}+i\gamma (A(z,t){\int }_{0}^{\infty }R(t^{\prime} ){|A(z,t-t^{\prime} )|}^{2}dt^{\prime} ),$$

where *A* is the electric field envelope, *β*_*k*_ are the dispersion coefficients at the central frequency *ω*_*0*_, *γ* is the Kerr nonlinearity coefficient. The response function $R(t)=(1-{f}_{R})\delta (t)+{f}_{R}{h}_{R}(t)$ includes both instantaneous electronic and delayed Raman contributions, where *f*_*R*_ represents the fractional contribution of the delayed Raman response to the instantaneous nonlinear polarization^37^.

### PCF parameters

**Figure 5:**
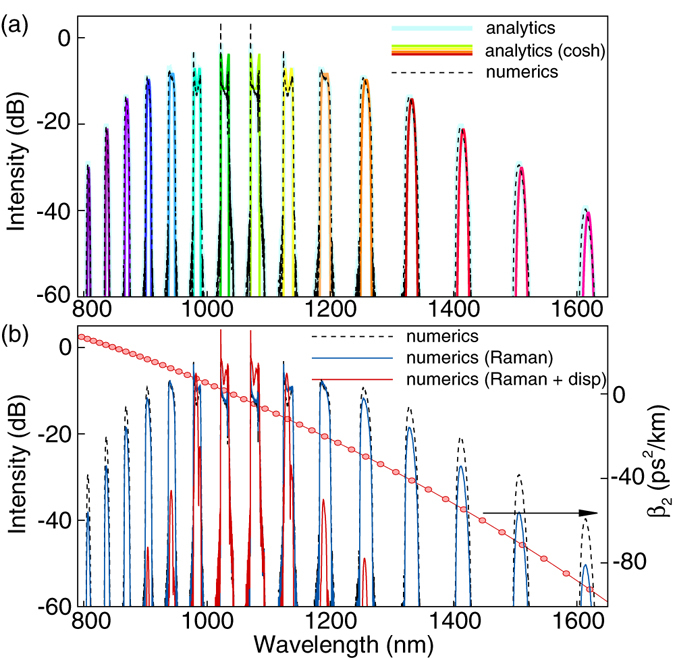
Parameters and results of calculations: Parameters and results of calculations for PCF SC-5.5-1040: output spectra calculated with analytical expressions (4) (blue line), or (5) (color line), and numerically (solution of the NLSE (6)) with neglected dispersion and Raman effects (**a**) and with account for Raman and dispersion (coefficient β_2_ is shown by circles) effects (**b**).

The output pulses were modeled analytically and numerically for the SC-5.5-1040 fiber (NKT Photonics Ltd.) with the parameters presented in Table 1, including Kerr nonlinearity (*γ*) and Raman gain (*g*_*R*_) coefficients, as well as dispersion coefficients associated with the Taylor series expansion of the propagation constant *β(ω)* near zero dispersion wavelength *λ*_*0*_. The full dispersion curve is shown in Fig. 5b together with the results of calculations.

**Table 1:** Parameters of PCF SC-5.5-1040.

Par	Value	Unit	Par	Value	Unit
*λ*_*0*_	1040	*nm*	*β*_*3*_	7.2417 × 10^−2^	*ps* ^3^/*km*
*ω*_*0*_	*2πc*/*λ*_*0*_	*rad*	*β*_*4*_	−1.0771 × 10^−4^	*ps* ^4^/*km*
*γ*	11	*W* ^−*1*^ *km* ^−*1*^	*β*_*5*_	1.3228 × 10^−7^	*ps* ^5^/*km*
*g*_*R*_	5.3	*W* ^−*1*^ *km* ^−*1*^	*β*_*6*_	−1.0321 × 10^−9^	*ps* ^6^/*km*
*β*_*2*_	0.670	*ps* ^2^/*km*	*β*_*7*_	7.5900 × 10^−12^	*ps* ^7^/*km*

### Results of calculations

The light blue line in Fig. 5a depicts the Fourier transform of the analytical solution (4), where *A(t)* has the form of a chirped DS, calculated numerically according to the model of the DS-RDS generator described in^18, 19^. The color line shows the output spectrum, calculated with formula (5), the corresponding parameters of the input pulse (hyperbolic cosine) are *P*_*0*_ = *|A*_*0*_ *|* ^2^ = 180 W, *T* = 15 ps, and *f* = 175. The dashed black line shows the numerical solution of the generalized NLSE obtained with the same initial condition as the analytical solution at negligible dispersion and Raman gain. Thus, Fig. 5a shows that the analytics work quite well in this case.

The blue line in Fig. 5b shows the solution of the generalized NLSE, where the Raman gain in PCF is taken into account. The red line refers to the case with both Raman gain and dispersion taken into account. One can see that dispersion could significantly decrease the number of spectral satellites (clones), generated at the DS-RDS propagation in a nonlinear fiber, while the influence of Raman effect is not so strong.

### DS/RDS generator

The DS/RDS generator (see Fig. 2) is based on a ring cavity consisting of a long (30 m) PM fiber (PMF), isolator (ISO), polarization controller (PC) and non-PM Yb-doped fiber amplifier (YDFA) enabling generation of a highly-chirped DS centered at 1025 nm. Inserting a delay line (DL) with a spectrally-selective PWDM splitter and a fiber coupler with a feedback coefficient R = 10^−4^ at the Stokes wavelength provides synchronous generation of a Stokes-shifted Raman DS at 1070 nm, according to the technique proposed in^18, 19^. The generated RDS leaves the cavity through the 99% port of the coupler, while the DS exits through the orthogonal-polarization port of the PBS.

### Characterization of the generated pulses

We have measured the spectral characteristics of the output pulses by an optical spectrum analyzer (Yokogawa 6370) and their temporal characteristics by the FROG (Mesaphotonics Ltd.) and intreferometric ACF (Avesta Ltd AA-20DD autocorrelator) techniques before and after a grating compressor. The FROG trace was retrieved by the VideoFROG Scan software. The pulse train was controlled by a 1 GHz photodiode (New Focus 1601FS-AC).
